# Nanoparticles Partially Restore Bacterial Susceptibility to Antibiotics

**DOI:** 10.3390/ma17071629

**Published:** 2024-04-02

**Authors:** Nina Bogdanchikova, Roberto Luna Vazquez-Gomez, Ekaterina Nefedova, Diana Garibo, Alexey Pestryakov, Evgenii Plotnikov, Nikolay N. Shkil

**Affiliations:** 1 Center for Nanoscience and Nanotechnology, National Autonomous University, Ensenada 22800, Mexico; nina@ens.cnyn.unam.mx (N.B.); dgaribo@ens.cnyn.unam.mx (D.G.); 2 School of Health Sciences, Autonomous University of Baja California, Ensenada 22890, Mexico; rluna@uabc.edu.mx; 3 Siberian Federal Scientific Centre of Agrobiotechnologies of the Russian Academy of Sciences, 630501 Novosibirsk, Russia; filll555@mail.ru (E.N.); nicola07@mail.ru (N.N.S.); 4 Research Institute by National Council of Science and Technology (CONACYT), Mexico City 03940, Mexico; 5 Research School of Chemistry and Applied Biomedical Sciences, Tomsk Polytechnic University, 634050 Tomsk, Russia; plotnikovev@tpu.ru

**Keywords:** silver nanoparticles, antibiotics, drug-resistance, partial recovery of susceptibility to antibiotics, cow mastitis

## Abstract

The growing resistance of bacteria to antibiotics is one of the main public health problems nowadays. The influence of silver nanoparticle (AgNP) pretreatment of 220 cows with mastitis on the susceptibility of *Staphylococcus epidermidis* bacteria to 31 antibiotics was studied. The obtained results were compared with the previous results for *Escherichia coli*, *Streptococcus dysgalactiae*, and *Staphylococcus aureus*. For all four bacteria, an increase in susceptibility (9.5–21.2%) to 31 antibiotics after cow treatment with AgNPs was revealed, while after first-line antibiotic drug treatment as expected, the susceptibility decreased (11.3–27.3%). These effects were explained by (1) the increase in the contribution of isolates with efflux effect after antibiotic treatments and its decrease after AgNP treatment and (2) the changes in bacteria adhesion and anti-lysozyme activity after these treatments. The effect of the increasing antibacterial activity of antibiotics after AgNP treatment was the most pronounced in the case of *E. coli* and was minimal in the case of *S. epidermidis*. With AgNP treatment, the time of recovery decreased by 26.8–48.4% compared to the time of recovery after treatment with the first-line antibiotic drugs. The AgNP treatment allows for achieving the partial restoration of the activity of antibiotics.

## 1. Introduction

Drug-resistant bacteria is a global health challenge caused by pathogens with strong resistance profiles. The spread of these pathogens becomes difficult to contain, which generates a negative impact on plant and animal health. Antimicrobial resistance (AMR) is the worldwide spread of pathogens through the food chain occurring due to the excessive overuse of antibiotics in the treatment of infections in animals that is contributing to the global resistome [1]. Therefore, there is an urgent need for the development of alternative, cost-effective, and efficient antimicrobial agents that can overcome antimicrobial resistance [2]. Some current alternatives to fight bacterial drug resistance include probiotics, bacteriophages, antimicrobial peptides, and plant-based substances, but these might have disadvantages such as low storage stability and high production costs [3]. Recently, silver nanoparticles (AgNPs) became one of the most promising antimicrobials for use in medicine thanks to their effective bactericidal activity. The unique properties of AgNPs, including particle size and morphological structure (the capacity to easily enter the bacterial cell wall and generate reactive oxygen species that ultimately lead to cell death), make them a promising alternative against infectious diseases [4]. Avoiding AMR propagation (via the food chain and treatment and control of livestock diseases) is crucial for livestock industries, safeguarding public health, animal production, food security, and food supply. Massive industrial breeding of cows and chickens makes a particularly high contribution to the propagation of AMR via the food chain. This is supported by the fact that cow milk produced worldwide for human consumption is about 81%, while buffalo, goat, sheep, and camel milk are 15, 2, 1%, and 0.5%, respectively [5].

Mastitis is one of the most frequent cow diseases, causing large economic losses due to reduced milk production during this illness [6]. Worldwide losses due to mastitis are approximately USD 35 billion annually [7]. The pathogens that are the major source of mastitis infection are mostly in the udder and enclosed areas. Around 137 microbial species (including bacteria and non-bacterial pathogens, such as fungi, yeasts, and mycoplasms) have been reported to be causative agents of bovine mastitis [8]. *Staphylococcus aureus*, *Escherichia coli*, *Streptococcus* spp., *Klebsiella* spp., and *Enterobacter* spp. are some of the most common bacteria associated with mastitis infection [9].

The Argovit-C^TM^ AgNP formulation has shown that its application to the treatment of cow mastitis can partially return 11–19.4% of the activity of 31 antibiotics towards three bacteria (*S. aureus*, *Str. dysgalactiae*, and *E. coli*), while treatments with first-line antibiotic drugs such as Lactobay, Spectromast LC, and Dienomast, in contrast, decreased activity of antibiotics by 23–27% [10,11,12]. The results of the study conducted on three bacteria are very promising because they open the possibility of contributing to solving the global problem of antibiotic resistance. However, the following question arises: Is the effect of “the AgNP treatment capacity to reduce bacterial resistance” revealed for three bacteria observed for all bacteria or only for some specific groups of bacteria? In our previous studies, it was revealed that this effect was observed for Gram-negative (*E. coli* [12]) and Gram-positive (*S. aureus* [10] and *Str. dysgalactiae* [11]) bacteria. Based on these results, can this phenomenon be extrapolated to all bacteria? Of course, no; there is a need for systematic investigation of this phenomenon on other bacteria. The longer the list of studied bacteria, the more the general nature of this phenomenon will be proven, and it will become clearer that it is applicable to all bacteria and not only to some specific bacteria. Therefore, an increase in the number of studied bacteria is of justified interest to expand the list of bacteria where this effect is observed or to find out the bacteria where this effect is either not observed or too low. Therefore, the present work is a continuation of our three previous works dedicated to the effect of AgNP treatment capacity to reduce bacterial resistance to 31 antibiotics [10,11,12] that will include the new Gram-positive *S. epidermidis* bacterium, which is also one of the main etiological agents of mastitis in dairy cows.

## 2. Materials and Methods

### 2.1. Sampling

Milk samples were collected from cows with mastitis before and after treatment with Lactobay and Argovit-C in livestock farms in the Novosibirsk region of the Russian Federation during 2018–2019. For milk sampling, udder teats were cleaned with a cotton swab moistened with 70% ethyl alcohol. A total volume of 10 mL of milk sample was collected into a sterile tube. All milk tubes were labeled with collection data and the number of cows. Milk samples were taken before and after applying the intracisternal treatment. Milk samples taken before treatment were considered as a control for every antibiotic. The samples were stored at a temperature of 8–10 °C until testing. Within 3–4 h, the samples were delivered for research.

### 2.2. Treatment Formulations

The experimental study involved 220 breeding farm cows with serous mastitis (approved by the Ethics Committee of the Siberian Federal Scientific Center for Agrobiotechnologies under Act No 00017, 25.02.2022). One cow group was treated with Lactobay (a first-line antibiotic-containing drug), and the second group was treated with Argovit™ AgNPs. Their general description is the following. 

LactobayTM, Norbrook Laboratories Limited (Newry, Northern Ireland, UK) is an antibacterial drug for intracisternal administration in the form of a suspension containing two antibiotics (1.5% sodium ampicillin and 4% sodium cloxacillin) as active ingredients. In veterinary medicine, the drug Lactobay is used as a first-line drug for the treatment of mastitis. The cows were injected intracisternally with Lactobay in a dose of 5 g with an interval of 12 h in accordance with the instructions of the manufacturer. The treatment was administered within 6 days until complete recovery (justified with the Biochemical California test).

Argovit-C^TM^, produced by Vector-Vita Scientific and Production Center, Novosibirsk, Russia, was provided by Dr. Vasily Burmistrov. Argovit-C, used for therapeutic and prophylactic purposes in the case of gastrointestinal diseases of calves, is a stable water suspension of silver nanoparticles (AgNPs) with a concentration of 200 mg/mL (20 wt.%). The metallic silver concentration is 12 mg/mL (1.2 wt.%) and the silver particle size is in the interval of 5–20 nm, with an average diameter of 15 nm. AgNPs are stabilized by polyvinylpyrrolidone (PVP) and hydrolyzed collagen with a total concentration of 18.8 wt.%. The remaining 80% of the weight corresponds to distilled water. Argovit-C was administered intracisternally to animals of the experimental group with a serous form of mastitis after 10-fold Argovit-C dilution (equivalent to 1.2 mg/mL of metallic Ag) at a dose of 10 mL once a day for 4 days until complete recovery, justified with the Biochemical California test. The characterization of the AgNP formulation is described in [13]. Briefly, the transmission electron microscopy data demonstrated that the AgNP sample contained separated spheroidal single particles with a size of 2–50 nm. A portion of the AgNPs produced aggregates with a size of about 100 nm. The UV–visible spectrum had a plasmon resonance peak with a wide maximum in the interval 420–460 nm. The hydrodynamic diameter varied between 30 and 350 nm, with an average value of 142.6 ± 0.241 nm, and the Zeta potential was +9.4 ± 6.28 mV. Because our experiments in vivo included 220 breeding farm cows with serous mastitis, the optimization of the doses and AgNP concentrations was carried out in the preliminary experiments. This optimization allowed us to achieve the maximum effectiveness of cow mastitis treatment and minimal consumption of silver-containing solution.

### 2.3. Isolation and Identification of S. epidermidis Bacteria

*S. epidermidis* bacteria were isolated from milk samples before and after the treatment (Lactobay or Argovit-C^TM^) of cows using a selective additive Staph-Strepto Supplement (HiMedia Laboratories Pvt. Ltd., Mumbai, India). The identification at the species level of the microbiota isolated from animals was carried out, considering the cultural, morphological, and biochemical properties of bacteria according to the generally accepted methods described in “The Bergey’s Manual of Determinative Bacteriology” [14].

### 2.4. Efflux Effect and Antimicrobial Sensitivity Testing

The disk diffusion method was used to determine the susceptibility of *S. epidermidis* to 31 antibiotics. The inhibition assay procedure was followed according to The Clinical and Laboratory Standards Institute (CLSI) [15]. All the tests were run in triplicate and the average result was taken.

For the efflux effect, *S. epidermidis* bacteria were cultured on Eugonic agar with ethidium bromide (1 mg/L). Samples were observed on a transilluminator after 24 h of incubation [16]. If the efflux effect is not present, the ethidium bromide penetrates into the bacterial cell, and the fluorescence is detected. In the presence of an efflux effect, no fluorescence is detected.

### 2.5. Statistical Analyses

Parametric and nonparametric analyses were used for statistical analysis using the STATISTICA 13.3 program (StatSoft Inc., Tulsa, OK, USA). The compilation, correction, systematization of the original information, and result visualization were processed with GraphPad Software 9.0, San Diego, CA, USA.

## 3. Results

### 3.1. The Number of Isolates of Bacteria

Table 1 presents the number of isolates of six bacteria measured in four bovine mastitis studies of our group. These studies were carried out at different times. Therefore, different bacteria species were predominant in every study. 

The maximum contributions of isolates of different bacteria were 22.5, 28.9, 43.3, and 37.2% for *S. aureus*, *Str. dysgalactiae*, *E. coli*, and *S. epidermidis*, respectively (bold in Table 1). The number of studied cows varied between 200 and 400 (Table 1). In the studies [10,11,12] and the present work, the bacterium with the maximum contribution was selected as the study focus. 

### 3.2. Change of Susceptibility to Antibiotics and Change of Isolate Contribution with Efflux Effect for S. epidermidis after Cow Treatment with the Antibiotic Drug and AgNPs

**Figure 1:**
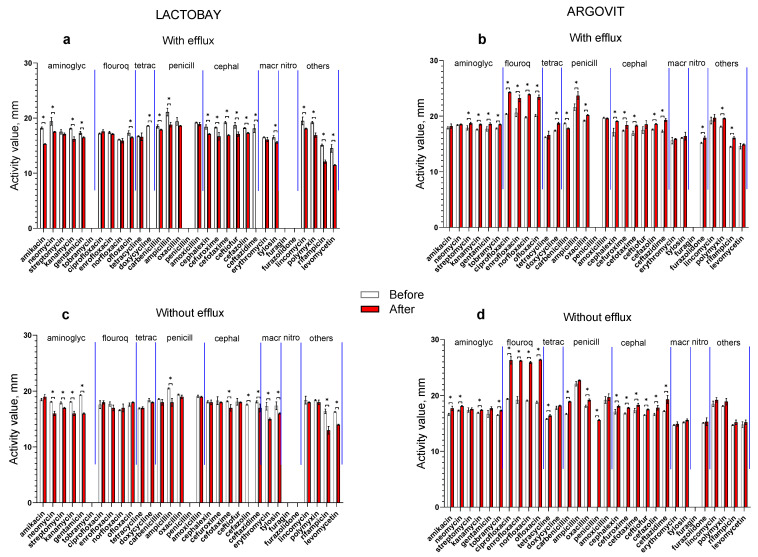
The activity of *S. epidermidis* before (white columns) and after (red columns) cow treatment with the antibiotic drug ((**a**)—isolates with efflux effect and (**c**)—isolates without efflux effect) and AgNPs ((**b**)—isolates with efflux effect and (**d**)—isolates without efflux effect). We marked the significant differences between the data with asterisks.

The susceptibility of *S. epidermidis* isolates to 31 antibiotics before and after treatment of cows suffering from mastitis with antibiotic drag (Lactobay) is presented in Figure 1a (for isolates with efflux effect) and Figure 1c (for isolates without efflux effect). Similar data after the treatment of cows with AgNPs is illustrated in Figure 1b (for isolates with efflux effect) and Figure 1d (for isolates without efflux effect). Figure 1 demonstrates that after antibiotic drug treatment, *S. epidermidis* susceptibility to 31 antibiotics in general decreases, while after treatment with AgNPs, it increases.

**Figure 2:**
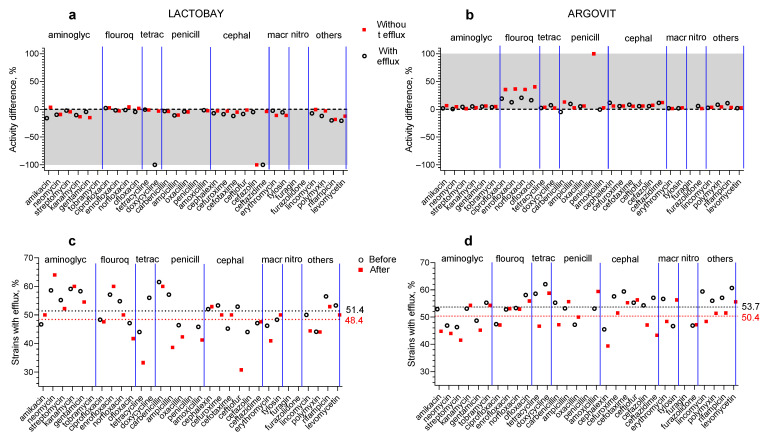
Relative susceptibility changes after cow treatment with the antibiotic drug (**a**) and AgNPs (**b**) for isolates with efflux effect (white circles) and without efflux effect (red squares). The shaded areas show the zone of change: negative for Lactobay and positive for AgNPs. The contribution of the number of isolates with efflux effect in the total number of isolates after cow treatment with Lactobay (**c**) and AgNPs (**d**): before the treatment—white circles, after treatment—red squares. Average values are marked with dotted lines of corresponding colors.

These differences can be seen more clearly in Figure 2a,b, where the relative differences (RDs) between sensitivity after treatment and before treatment are presented for both antibiotic and AgNP treatments and for isolates with and without the efflux effect. The RD was calculated according to the following formula:

RD = (S_after_ − S_before_) × 100%/S_before_,

where S_after_ and S_before_ are average values of susceptibilities after and before treatment, respectively. The shaded zones clearly demonstrate that the RD values are predominantly negative for antibiotic drug treatment (Figure 2a) and positive for AgNP treatment (Figure 2b). Figure 2c,d show the contribution of the number of isolates with the efflux effect in the total number of isolates after cow treatment with the antibiotic drug (Figure 2c) and AgNPs (Figure 2d). Data before the treatment are represented by white circles and after treatments by red squares. The average values marked in Figure 2c,d with dotted lines with corresponding colors show that after both treatments (antibiotic drug treatment and AgNPs) the contribution decreases by only three percent. 

So, the obtained results for both formulations practically show the absence of change in the contributions of the number of *S. epidermidis* isolates with the efflux effect. For other bacteria studied earlier, these differences were much higher, and this will be discussed below.

## 4. Discussion

### 4.1. Susceptibility Change

In this part of the article, we compare and analyze the results obtained for four bacteria.

**Figure 3:**
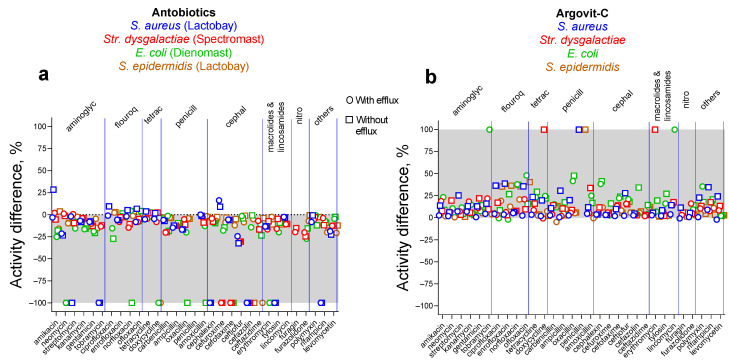
The relative activity changes after treatment of cow mastitis with antibiotics (**a**) and AgNPs (**b**). The shaded areas show the zone of change: a negative zone for antibiotic drugs and a positive zone for AgNPs. Data for *S. aureus* are depicted with blue symbols, *Str. dysgalactiae* with red symbols, *E. coli* with green symbols, and *S. epidermidis* with brown symbols.

The comparison of the results for *S. aureus* (blue symbols), *Str. dysgalactiae* (red symbols), *E. coli* (green symbols) (obtained by our group earlier [10,11,12]), and *S. epidermidis* (brown symbols) obtained in the present work are presented in Figure 3. 

As seen from the shaded zones of Figure 3a,b, in general, for isolates with and without efflux, the susceptibility to 31 antibiotics after treatment with antibiotic drugs decreased, but it increased after treatment with AgNPs. Table 2 and Table 3 present data on average relative changes of susceptibility for four studied bacteria to 31 antibiotics after treatment with antibiotic drugs and AgNPs, respectively. 

After the treatment with antibiotic drugs, the susceptibility of four bacteria to most antibiotics (for isolates with and without efflux effect) decreased by 11.3–27.3% (Table 2), while after AgNP treatment it increased by 9.5–21.2% (Table 3). The total increase in antibiotic activity (when AgNPs are used instead of antibiotic drugs) is 20.8–48.5% (Table 4).

Table 5 and Table 6 present a more detailed description of changes for 31 antibiotics, including the number of isolates with increasing, decreasing, and unchanged susceptibility after antibiotic drug treatment or AgNP treatments, respectively. Data for isolates with and without efflux for four bacteria show that after antibiotic treatment, for 28 isolates, the activity of antibiotics remained absent; for 30 isolates, the activity completely disappeared; for 167 isolates, it decreased, and only for 22 isolates did it increase (Table 5). 

A very different data arrangement is observed after AgNP treatment. Antibiotic activity remained absent only for 11 isolates (that is 2.5 times less than for antibiotics), and no loss of activity was observed for any isolate, and vice versa, the antibiotic activity appeared for 8 isolates (after antibiotic treatment none of the antibiotics showed the appearance of activity), and activity increased for 220 isolates and decreased only for 7 (Table 6). So, we can say that after antibiotic treatment, 23 positive changes (susceptibility increase) and 225 negative changes (susceptibility decrease) were observed, so positive changes represent only 9.2% of the changes (Table 5). 

However, after AgNP treatment, 228 positive changes and 18 negative changes occurred, where positive changes represent 92.7% of the total changes (Table 6). These data (Table 5 and Table 6) detail the merits shown for the susceptibility of using AgNP treatments instead of antibiotic treatments, as presented in Table 2. 

### 4.2. Change in the Percentage of Isolates with Efflux Effect

**Figure 4:**
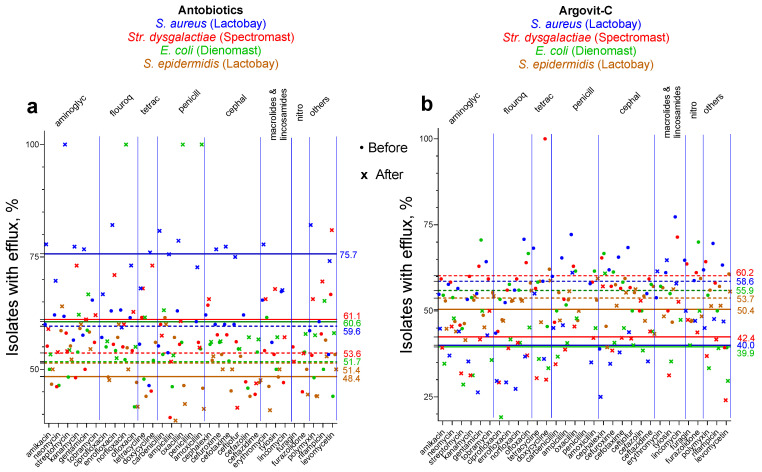
Change of the contribution in the number of isolates with efflux effect after antibiotic treatments (**a**) and AgNP treatments (**b**). Average values before treatments are marked with dotted lines, and after treatments with solid lines. Data for *S. aureus* are depicted with blue symbols, *Str. dysgalactiae* with red symbols, *E. coli* with green symbols, and *S. epidermidis* with brown symbols.

Figure 4 and Table 7 present the change in the percentage of the number of isolates with the efflux effect after antibiotic treatments and AgNP treatments. 

In Figure 4, the average values before treatments are marked with dotted lines and after those treatments are marked with solid lines. For *S. aureus*, *Str. dysgalactiae*, and *E. coli,* after AgNP treatment the percentage of isolates with the efflux effect decreased by 16.0–18.6%, while after treatment with antibiotic drugs, it increased by 8.9–17.5%. Unexpectedly, for *S. epidermidis*, changes were very small for antibiotic and AgNP treatments (−3.0 and −3.3%, respectively). This indicates that probably in this bacteria efflux pumps do not play an important role in changing the susceptibility to antibiotics by AgNP treatment. This can explain the fact that for *S. epidermidis,* the total increase in antibiotic activity when AgNPs are used instead of antibiotic drugs is only 20.8%, while for the other three bacteria, the total increase is 32.3–48.5% (Table 4). For three bacteria (*S. aureus*, *Str. dysgalactiae*, and *E. coli*), the significant decrease in the percentage of isolates with the efflux effect after AgNP treatment explains the substantial increase in the susceptibility to antibiotics. But for *S. epidermidis*, there is probably no efflux pump activation or deactivation, although other virulence factors play an important role in the influence of AgNPs on susceptibility to antibiotics. In our previous work, it was shown that the anti-lysozyme activity of five antibiotics for *S. epidermidis* ATCC 14990 was 100%, but cultivation with AgNPs decreased the anti-lysozyme activity by 20%, which was a greater decrease than for the other six bacteria [17]. The highest decrease in the adhesion index was also observed for *S. epidermidis* after AgNP treatments [17]. Probably for *S. epidermidis* changes caused by AgNPs are not connected to efflux pumps but are rather connected to changes in the bacteria adhesion index and its anti-lysozyme activity.

### 4.3. Change in the Number of Isolates of Four Bacteria after Treatments 

**Figure 5:**
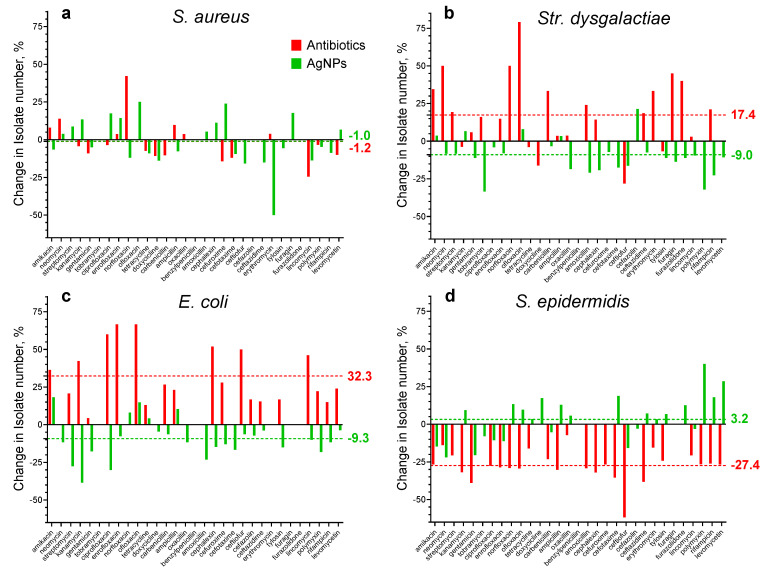
The percentage of the change in the number of bacteria isolates after treatments with antibiotic drugs (red bars) and AgNPs (green bars) for four studied bacteria. Average values are marked with dotted lines of the corresponding color; (**a**), (**b**), (**c**), and (**d**) represent data for *S. aureus*, *Str. dysgalactiae*, *E. coli*, and *S. epidermidis*, respectively.

The percentage of the change in the number of isolates (where bacteria were detected, but on the levels of healthy cows) after treatments with antibiotic drugs (red bars) and AgNPs (green bars) for the four studied bacteria are presented in Figure 5 and Table 8. 

For *S. aureus*, no correlation with the treatment type was observed and, in both cases, the average value of the number of isolates after treatments practically did not change because the difference is only about one percent (Table 8). However, for some antibiotics, the differences reached almost 50%. After antibiotic treatment, the average number of isolates of *Str. dysgalactiae* and *E. coli* increased by 17.4 and 32.3%, respectively. After AgNP treatment, in contrast, they decreased by 9.0 and 9.3%, respectively. After antibiotic treatment, the average number of *S. epidermidis* isolates decreased by 27.4%, while after AgNP treatment it practically did not change (the difference is only 3.2%). 

These results show that after AgNP treatment, the average number of isolates of the four studied bacteria does not change or decrease, while after antibiotic treatment it is without change for *S. aureus*, increased for *Str. dysgalactiae* and *E. coli* isolates (by 17.4 and 32.3%, respectively), and decreased for *S. epidermidis* isolates (27.4%). To understand the differences between the four bacteria behaviors, further experiments are necessary. However, it is worth mentioning that the behavior of *S. epidermidis* is very different from other bacteria.

### 4.4. Cow Recovery Period

Table 9 presents the duration of the treatments with antibiotic drugs and with AgNPs and the percentage of acceleration of AgNP treatment compared with antibiotic drug treatment. 

The cow recovered after 4.8–6.7 days of treatment with antibiotic drugs and after 2.9–4.9 days of AgNP treatment (Table 9). 

With the application of AgNPs, the cow recovery period was reduced by 26.8–48.4% (Table 9). This is an additional advantage of the application of the AgNP formulation instead of antibiotic drugs. The maximum decrease in the recovery period was observed for *E. coli* (48.4%), and the minimum one for *S. epidermidis* (26.9%) and *S. aureus* (26.8%). 

Table 10 illustrates that *E. coli* demonstrated maximum benefits of AgNP use in relation to (1) a growth of susceptibility to antibiotics, (2) a decrease in the bacteria isolate number, and (3) a decrease in the treatment duration. For *S. epidermidis*, on the contrary, minimum effectiveness from AgNP use was observed. 

Maximum effects were observed for Gram-negative bacteria (*E. coli*), and effects for Gram-positive bacteria were more moderate. In the future, more bacteria should be studied to confirm this tendency.

### 4.5. New Approach to Reducing Bacterial Drug Resistance 

It is important to highlight that a series of our works presents a new approach to studying the influence of AgNPs on bacterial drug resistance [10,11,12,17]. In the literature, the combined application of antibiotics and nanoparticles (especially silver nanoparticles) aiming to increase their activity against pathogenic microorganisms is widely discussed [18]. The combined application sometimes leads to synergetic effects but sometimes to neutral or antagonistic effects. The cases of combined application of AgNPs with optimal antibiotics leading to synergetic effects help to fight multidrug-resistant bacteria. Our new approach (described in a series of our previous publications [10,11,12,17] and in the present work) consists of a non-simultaneous use of AgNPs and antibiotics, applying AgNPs in a pretreatment stage followed by the antibiotic treatment stage.

Pretreatment (preliminary treatment) is an action that aims to improve the results of the following treatment. It is very widely used in science and in practice. Below we will give some clear and simple examples. In the textile industry, pretreatment is used for the elimination of unwanted compounds from fabrics. In the water purification process, the pretreatment presents the stage of the elimination of oil, sand, and grease. Pretreatment with a salt solution on the street helps to melt snow, which makes it easier to clean streets from snow. To maintain catalysts in an active state in chemical plants, the catalysts are pretreated in different atmospheres [19]. Pretreatments are widely used in medicine too. For example, pretreatment with the pyridostigmine bromide drug was used in the Gulf War to protect against complications from soman, the chemical warfare nerve agent [20], and pretreatment with mifepristone can be used to prevent miscarriage [21]. Historically, the influence of AgNPs on the activity of antibiotics was carried out when they were applied together. Therefore, we did not find any article (published before the series of our work on this topic [10,11,12,17]) dedicated to the influence of animal pre-treatment with AgNPs on bacterial resistance to antibiotics. The results of the series of our works revealed that animal pre-treatment with AgNPs led to a decrease in the resistance of bacteria to antibiotics. This revealed phenomenon merits further meticulous study due to the nowadays great importance of the bacterial drug resistance problem in public health.

## 5. Conclusions

In the present work, the influence of AgNP pretreatment of 220 cows with mastitis on the susceptibility of *S. epidermidis* bacteria to 31 antibiotics was studied. The observed results showed that after cow treatment with the first-line antibiotic drug Lactobay, susceptibility to 31 antibiotics decreased on average by 11.3%, while after AgNP treatment it increased on average by 9.5%. These results are analogous to the results obtained by our group earlier for *E. coli*, *Str. dysgalactiae*, and *S aureus.* For these bacteria, the antibiotic susceptibility after antibiotic treatment decreased by 19.2–27.3%, and after AgNP treatment it increased by 11.4–21.2% [12]. These effects were explained by (1) the increase in the contribution of isolates with the efflux effect after antibiotic treatments and its decrease after AgNP treatment and/or (2) the changes in bacteria adhesion and anti-lysozyme activity after these treatments. This work presents a new approach to reducing bacterial drug resistance using pretreatment with AgNPs, which differs from the previous approach based on the use of the combined application of antibiotics together with AgNPs. The obtained results open new perspectives for AgNP application for recovering the antibiotic activities decreased due to bacterial resistance. On the other hand, the present study is translational research because its results can be applied directly in practice. They were obtained from 200–400 breeding farm cows with serious mastitis in vivo fieldwork. With AgNP treatment, the recovery period decreased by 26.8–48.4% compared to the treatment with the first-line antibiotic drugs. Moreover, after AgNP treatment, the bacteria became more sensitive to antibiotics and cows could be treated with fewer antibiotic doses. 

## Figures and Tables

**Table 1:** The number of isolates of six bacteria measured in four bovine mastitis studies of our group.

Bacteria	The Number and Percent of Isolates of Six Bacteria Measured in Four Studies of Our Group			
	*S. aureus* Study, 400 Cows [1]	*Str. dysgalactiae* Study, 300 Cows [2]	*E. coli* Study, 200 Cows [3]	*S. epidermidis* Study, 220 Cows (Present Work)
* Str. dysgalactiae *	49.5 (12.4%)	**86.6 (28.9%)**	43 (21.5%)	30.9 (14.1%)
* S. aureus *	**90 (22.5%)**	73.3 (24.4%)	60 (30%)	74.5 (33.9%)
* Str. pyogenes *	40 (10%)	50.0 (16.7%)	32.5 (16.3%)	36.4 (16.5%)
* E. coli *	12.7 (3.2%)	16.7 (5.6%)	**86.5 (43.3%)**	19.1 (8.7%)
* S. epidermidis *	55 (13.8%)	32 (10.7%)	37 (18.5%)	**81.8 (37.2%)**
* Str. agalactiae *	45 (11.3%)	26.7 (8.9%)	33.5 (16.8%)	28.6 (13.0%)

**Table 2:** The average relative change in susceptibility of four studied bacteria to 31 antibiotics after antibiotic drug treatment.

Bacteria	Isolates without Efflux Effect		Isolates with Efflux Effect		Isolates with Activity Change	
	Number of Antibiotics	Average Change in Susceptibility	Number of Antibiotics	Average Change in Susceptibility	Number of Antibiotics	Average Change in Susceptibility
* S. aureus *	27	−25.8%	27	−24.5%	54	−25.1%
* Str. dysgalactiae *	29	−19.2%	29	−19.2%	58	−19.2%
* E. coli *	27	−30.2%	27	−24.4%	54	−27.3%
* S. epidermidis *	27	−8.50%	27	−14.0%	54	−11.3%

**Table 3:** The average relative change in susceptibility of four studied bacteria to 31 antibiotics after AgNP treatment.

Bacteria	Isolates without Efflux Effect		Isolates with Efflux Effect		Isolates with Activity Change	
	Number of Antibiotics	Average Change in Susceptibility	Number of Antibiotics	Average Change in Susceptibility	Number of Antibiotics	Average Change in Susceptibility
* S. aureus *	30	+19.9%	29	+2.9%	59	+11.4%
* Str. dysgalactiae *	30	+17.2%	29	+8.9%	59	+13.1%
* E. coli *	29	+19.4%	29	+22.9%	58	+21.2%
* S. epidermidis *	30	+12.3%	29	+6.60%	59	+9.5%

**Table 4:** The total increase in antibiotic activity when AgNPs are used instead of antibiotic drugs.

Bacteria	The Total Increase in Antibiotic Activity When AgNPs Are Used Instead of Antibiotic Drugs
* S. aureus *	36.5%
* Str. dysgalactiae *	32.3%
* E. coli *	48.5%
* S. epidermidis *	20.8%

**Table 5:** The number of isolates with increasing, decreasing, and unchanged susceptibility after antibiotic drug treatments for four bacteria.

Bacteria	Number of Isolates: Without Efflux/with Efflux for Each Case					
	Activity Remains Absent	Activity Disappeared (−100%)	Activity Appeared (+100%)	Activity Decreased (−Δ%)	Activity Increased (+Δ%)	Activity Constant (Δ = 0%)
*S. aureus*	4/4	6/5	0/0	14/20	7/1	0/1
*Str. dysgalactiae*	2/2	3/3	0/0	24/23	2/3	0/0
*E. coli*	4/4	6/4	0/0	18/22	3/1	0/0
*S. epidermidis*	4/4	1/2	0/0	22/24	4/1	0/0
Data for four bacteria	14/14	16/14	0/0	78/89	16/6	0/1
Data for isolates with and without efflux for four bacteria	28	30	0	167	22	1
Overall change	23 positive changes * and 225 negative changes **, where positive changes represent 9.2%					

* Positive changes correspond to the susceptibility increase. ** Negative changes correspond to susceptibility decrease.

**Table 6:** The number of isolates with increasing, decreasing, and unchanged susceptibility after AgNP treatments for four bacteria.

Bacteria	Number of Isolates: Without Efflux/With Efflux for Each Case					
	Activity Remains Absent	Activity Disappeared (−100%)	Activity Appeared (+100%)	Activity Decreased (−Δ%)	Activity Increased (+Δ%)	Activity Constant (Δ = 0%)
*S. aureus*	1/2	0/0	1/0	0/2	29/27	0/0
*Str. dysgalactiae*	1/2	0/0	2/0	0/1	28/28	0/0
*E. coli*	2/2	0/0	2/2	0/2	27/25	0/0
*S. epidermidis*	1/2	0/0	1/0	0/2	29/27	0/0
Data for four bacteria	5/6	0/0	6/2	0/7	113/107	0/0
Data for isolates with and without efflux for four bacteria	11	0	8	7	220	0
Overall change	228 positive changes * and 18 negative changes **, where positive changes represent 92.7%					

* Positive changes correspond to the susceptibility increase. ** Negative changes correspond to susceptibility decrease.

**Table 7:** Change in the percentage of isolates with efflux effect after treatments with antibiotic drugs and AgNPs for four bacteria.

Bacteria	Percentage of Isolates with Efflux Effect						The Increase in Antibiotic Activity When AgNP Treatment Is Used Instead of Antibiotic Treatment, %
	Antibiotic Treatment			AgNP Treatment			
	Before Treatment, %	After Treatment, %	Difference, %	Before Treatment, %	After Treatment, %	Difference, %	
* S. aureus *	59.6	75.7	+16.1	58.6	40.0	−18.6	34.7
* Str. dysgalactiae *	53.6	61.1	+17.5	60.2	42.4	−17.8	35.3
* E. coli *	51.7	60.6	+8.9	55.9	39.9	−16.0	24.9
* S. epidermidis *	51.4	48.4	−3.0	53.7	50.4	−3.3	0.3

**Table 8:** The average number of bacteria isolates of four studied bacteria before and after treatments with antibiotic drugs and AgNPs.

Bacteria	The Change in the Average Value of the Number of Isolates		Benefit from AgNP Treatment Compared to Antibiotic Drug Treatments, %
	After Antibiotic Treatment, %	After AgNP Treatment, %	
* S. aureus *	−1.2	−1.0	−0.2
* Str. dysgalactiae *	+17.4	−9.0	26.4
* E. coli *	+32.3	−9.3	41.6
* S. epidermidis *	−27.4	+3.2	−30.6

**Table 9:** Duration of the treatment of serous mastitis with antibiotic drugs and with AgNPs and percentage of reduction of recovery period with AgNP treatment compared with antibiotic drug treatment.

Bacteria	Antibiotic Drug Name	Duration of Treatment with Antibiotic Drugs, Days	Duration of Treatment with AgNPs, Days	Acceleration of Treatment with AgNPs Compared to Antibiotic Treatment, %
*S. aureus*	Lactobay	5.6 ± 0.4	4.1 ± 0.4	26.78
*Str. dysgalactiae*	Spectromast	4.8 ± 0.2	2.9 ± 0.1	39.58
*E. coli*	Dienomast	6.2 ± 0.2	3.2 ± 0.3	48.38
*S. epidermidis*	Lactobay	6.7 ± 0.2	4.9 ± 0.3	26.86

**Table 10:** Advantages of application of AgNPs instead of antibiotic-containing drugs for cow mastitis treatment.

Type of Advantage	Change Interval	Effectivity Order	Maximum Effectiveness	Minimum Effectiveness
Susceptibility increase	20.8–48.5%	*E. coli *> * S. aureus *> * Str. dysgalactiae *> * S. epidermidis*	*E. coli*, 48.5%	*S. epidermidis*, 20.8%
Increase in the percentage of isolates without efflux effect	0.3–35.3%	*Str. dysgalactiae~S. aureus *> * E. coli* > *S. epidermidis*	*S. dysgalactiae* ~ *S. aureus*, 35%	*S. epidermidis*, 0.3%
Decrease in bacteria isolate number	−30.6–+41.6% *	*E. coli *> * Str. dysgalactiae *> * S aureus *> * S. epidermidis*	*E. coli*, 41.6%	*S. epidermidis*, −30.6% *
Treatment duration decrease	26.4–48.4%	*E. coli* > *Str. dysgalactiae *> * S. epidermidis ~ S aureus E. coli* > *Str. dysgalactiae *> * S. epidermidis ~ S. aureus*	*E. coli*, 48.4%	*S. epidermidis* and *S. aureus*, 26.4%

* Unlike the other three bacteria for *S. epidermidis* the advantage in decrease of bacteria isolate number was observed after antibiotic drug treatment, not after AgNP treatment.

## Data Availability

Data are contained within the article.
